# Exploiting Connections for Viral Replication

**DOI:** 10.3389/fcell.2021.640456

**Published:** 2021-03-18

**Authors:** Louise H. Wong, James R. Edgar, Andrea Martello, Brian J. Ferguson, Emily R. Eden

**Affiliations:** ^1^UCL Institute of Ophthalmology, London, United Kingdom; ^2^Department of Pathology, University of Cambridge, Cambridge, United Kingdom

**Keywords:** Membrane contact sites (MCS), double membrane vesicles (DMVs), SARS-CoV-2, viral replication, lipid transport

## Abstract

Severe acute respiratory syndrome coronavirus 2 (SARS-CoV-2), the cause of the COVID-19 (coronavirus disease 2019) pandemic, is a positive strand RNA (+RNA) virus. Like other +RNA viruses, SARS-CoV-2 is dependent on host cell metabolic machinery to survive and replicate, remodeling cellular membranes to generate sites of viral replication. Viral RNA-containing double-membrane vesicles (DMVs) are a striking feature of +RNA viral replication and are abundant in SARS-CoV-2–infected cells. Their generation involves rewiring of host lipid metabolism, including lipid biosynthetic pathways. Viruses can also redirect lipids from host cell organelles; lipid exchange at membrane contact sites, where the membranes of adjacent organelles are in close apposition, has been implicated in the replication of several +RNA viruses. Here we review current understanding of DMV biogenesis. With a focus on the exploitation of contact site machinery by +RNA viruses to generate replication organelles, we discuss evidence that similar mechanisms support SARS-CoV-2 replication, protecting its RNA from the host cell immune response.

## Introduction

**FIGURE 1:**
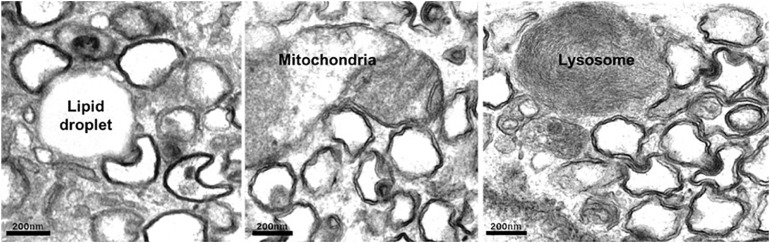
Human lung carcinoma epithelial cell (A549) infected with SARS-Co-V-2 were fixed and prepared for electron microscopy. Abundant DMVs are visible, often in contact with host cell organelles. Scale bar, 200 nm.

On entry into the host cell, the viral genome is released, and replication–transcription complexes (RTCs) are assembled that drive viral genome replication and expression of viral proteins. Replication of +RNA virus takes place in the cytoplasm, potentially exposing viral RNA to host cell defense mechanisms. However, viruses have developed creative ways to circumvent the host’s defensive response. RTCs assemble in association with cytoplasmic membranes, and +RNA viruses co-opt host factors to induce extensive membrane remodeling including the formation of double-membrane vesicles (DMVs) (Wolff et al., 2020b). This rearrangement of cellular membranes provides structural scaffolding for viral RTCs, as well as protection from antiviral host responses. DMVs are clearly visible in cells infected with a variety of +RNA viruses, including severe acute respiratory syndrome coronavirus 2 (SARS-CoV-2) (Figure 1). As the name suggests, DMVs are small vesicles, of approximately 100–300 nm in diameter, surrounded by two membranes, often clustered together. DMVs are major replication organelles (ROs), housing viral RNA that is enriched in the DMV core (Knoops et al., 2008; Klein et al., 2020). A molecular pore has been described spanning both membranes, providing a transport route for viral RNA out of the DMVs to be translated and packaged (Wolff et al., 2020a).

The primary targets for SARS-CoV-2 infection are cells of the nasal and respiratory epithelium, which form polarized monolayers, with distinct apical and basolateral domains. The polarized distribution of receptors and proteases may impact mechanistically on viral replication. Primary respiratory epithelial cells offer the most accurate reflection of SARS-CoV-2 target tissues, but epithelial cell lines have proved extremely valuable in informing current understanding of coronavirus cell biology. For example, much has been learned from viral infection of the widely used African green monkey kidney epithelial cell line, Vero-E6. However, although Vero-E6 cells express the SARS-CoV-2 receptor ACE2 on their apical surface, they do not express the serine protease TMPRSS2 that activates viral spike protein (Hoffmann M. et al., 2020). The human lung adenocarcinoma Calu-3 cells are perhaps a more relevant cell line, expressing both ACE2 and TMPRSS2, all be it at higher and lower levels, respectively, than human lung tissue (Kawase et al., 2012), but the influence of cell type on viral behavior, or the relationship between viral entry and biogenesis of the RO, is not yet clear.

## DMV Biogenesis

Although our understanding of the SARS-CoV-2 DMV molecular architecture is incomplete, studies on other coronaviruses, SARS-CoV and Middle East respiratory syndrome–related coronavirus (MERS), have shown that viral non-structural protein (nsp)s play a key role in DMV formation. Interaction between the luminal loops of SARS-CoV nsp3 and nsp4 was shown to drive membrane rearrangements (Hagemeijer et al., 2014), and expression of these two nsps is sufficient to induce DMV formation (Angelini et al., 2013; Oudshoorn et al., 2017). DMVs have an interesting topology; studies in Vero-E6 cells have revealed a cytosolic core, whereas the lumen of the endoplasmic reticulum (ER) appears continuous with the material between the inner and outer DMV membranes, indicating that DMVs are derived from host ER membrane (Knoops et al., 2008). However, markers from other organelles, including Golgi, have also been identified on ROs (Schlegel et al., 1996). Coxsackie B virus 3 forms their ROs from both ER and Golgi membranes. The earliest ROs formed are single-membrane tubules, which are transformed into DMVs and multilamellar vesicles as infection progresses (Limpens et al., 2011). By electron tomography, early viral ROs, which later transform into DMVs, appear to originate from *cis*-Golgi membranes (Belov et al., 2012). Moreover, newly synthesized viral RNA was detected at the *trans*-Golgi network (TGN) in coxsackievirus B3–infected cells, and later-stage ROs remained positive for the GTPase Arf1, which in uninfected cells, localizes to the TGN (Hsu et al., 2010).

Machine learning models indicate that SARS-CoV-2 viral RNA also localizes to the mitochondria (Wu et al., 2020), raising the possibility that SARS-CoV-2 may also manipulate mitochondrial membrane to generate mitochondrial-derived DMVs. Interestingly, point mutations in murine coronavirus that decrease the number of ER-derived DMVs also resulted in relocalization of nsp3 and nsp4 to the mitochondria (Clementz et al., 2008). RNA replication of other +RNA viruses, such as alphanodavirus flock house virus, has been shown to occur on the outer mitochondrial membranes of infected cells (Miller et al., 2001). Mitochondrial-derived vesicles (MDVs), of approximately 70–150 nm, can extrude from mitochondrial membranes to transport mitochondrial protein and lipids to other organelles (Sugiura et al., 2014). Although the contribution of MDVs to RO formation has not yet been established, it has been suggested that mitochondrial damage by SARS-CoV-2 could induce the generation of double-membrane, viral RNA-containing MDVs (Singh et al., 2020).

Like mitochondria, autophagosomes are enclosed by a characteristic double-membrane and have also been implicated in coronavirus RO biogenesis. In SARS-CoV–infected Vero-E6 cells, the viral replicase protein, nsp8, colocalized with the autophagosome marker LC3 (Prentice et al., 2004b). Similarly, in cells infected with mouse hepatitis virus (MHV), two replication complex–localized viral proteins (p22 and N) also colocalized with LC3, as well as ATG12, which with ATG5 promotes LC3 lipidation, an important step in autophagosome formation. MHV replication was impaired in autophagy-deficient cells and rescued by ATG5 re-expression (Prentice et al., 2004a). In contrast, although confirming the overlap between SARS-CoV viral replicase proteins and LC3, another study found that neither ATG5 nor LC3 lipidation was required for MHV replication (Zhao et al., 2007). Likewise, viral titers did not differ between SARS-CoV–infected wild-type or ATG5^–/–^ mouse embryonic fibroblasts (Schneider et al., 2012). Careful analysis of LC3-positive DMVs revealed that only the endogenous non-lipidated LC3-I associates with MHV-induced DMVs, and while the LC3 lipidation machinery was dispensable for MHV replication, decreased LC3 impaired MHV replication (Reggiori et al., 2010). Consistent with viral RO assembly being facilitated by autophagosome formation, but not lysosomal targeting and fusion, a recent study reported an incomplete autophagy response to SARS-CoV-2 infection (Qu et al., 2020). Expression of SARS-CoV-2, but not SARS-CoV, ORF3a, was sufficient to trigger incomplete autophagy, where autophagosome formation is increased, but maturation impaired. Importantly, inhibition of autophagosome formation reduced viral replication. Similarly, MERS-CoV infection increased the number of phagocytic vesicles, but impaired autophagosome–lysosome fusion. However, induction of the complete autophagy process severely impaired MERS-CoV replication (Gassen et al., 2019). A genome-wide study performed in a human hepatoma cell line (Huh-7.5) expressing ACE2/TMPRSS2 and validated in human pulmonary epithelial A549 cells expressing ACE2/TMPRSS2 identified TMEM41B as an essential host factor for SARS-CoV-2 and three seasonal CoVs (HCoV-OC43, HCoV-NL63, and HCoV-229E) (Schneider et al., 2020). Similarly, TMEM41B was also recently found to be essential for flavivirus replication (Hoffmann et al., 2021). TMEM41B is an ER resident protein with known roles in phagophore maturation (Morita et al., 2018; Shoemaker et al., 2019) and lipid mobilization (Moretti et al., 2018). As in both studies no other autophagy-related proteins scored as positive regulators of viral infection, it is possible that TMEM41B’s role in lipid mobilization and membrane remodeling, rather than phagophore maturation, is more relevant for virus infectivity. Taken together, these findings suggest that coronaviruses can exploit autophagosome formation machinery to support DMV biogenesis, while stalling lysosome fusion to evade autophagy-mediated degradation.

DMV formation occurs early in infection. Within 2 h postinfection (hpi) with SARS-CoV, DMVs are found throughout the cytoplasm, occasionally connected to ER membrane, consistent with the ER being the source of DMV membranes. At the start of infection, the inner and outer DMV membranes are tightly apposed, but the distance between the two membranes becomes less uniform, with wider gaps evident at later stages. Within 4 hpi, DMVs are dramatically increased in number, often in clusters of up to 300 vesicles at 7 hpi. At late stages, membranous vesicle “packets” arise that appear as a single membrane surrounding one or several single-membrane vesicles, possibly as a result of DMV outer membranes fusing together (Harak and Lohmann, 2015).

## Hijacking Lipid Biosynthetic Pathways

Lipid composition has a strong influence on the physical properties of cellular membranes, including fluidity and curvature, as well as the activity of membrane proteins. As such, different organelles have a specialized lipid composition according to their specific needs. Cholesterol is an essential membrane lipid that maintains membrane integrity but is heterogeneously distributed throughout cellular membranes (Maxfield and Menon, 2006). As cholesterol levels are relatively low at its site of synthesis (the ER), cholesterol transport to other membranes tends to occur against its concentration gradient and utilizes a lipid counter-exchange mechanism at sites of contact between the ER and other organelles (see Box 1).

Interesting differences in lipid composition between viral DMVs and the ER membranes from which they are derived have been uncovered. Hepatitis C virus (HCV)–induced DMVs contain nine times more cholesterol than the host ER membranes (Paul et al., 2013) and are also enriched in the phospholipid PI4P (Reiss et al., 2011). Thus, viral induction of host lipid synthesis is likely required to supply the lipids necessary for the membrane rearrangements associated with viral replication (Li et al., 2007). Recently, a focused interactome CRISPR screen identified a dependency for host factors involved in cholesterol biosynthesis for coronaviruses including SARS-CoV-2 (Hoffmann H.-H. et al., 2020). Host lipid biosynthetic pathways are largely regulated by sterol regulatory element binding proteins (SREBPs). Inhibition of SREBP-mediated transcription was found to suppress viral replication in a variety of MERS-CoV–infected cells including primary small epithelial cells, as well as increasing survival rates/reducing alveolar damage in infected mice (Yuan et al., 2019). A lipodomics study on human coronavirus 229E-infected cells found that total cellular lipid composition is also altered, with a global increase in fatty acids and glycerophospholipid (Yan et al., 2019). It is likely that these lipids supply membrane for the generation of viral ROs. Enrichment of phosphatidyl choline at viral DMV is also observed in other +RNA viruses such as HCV (Zhang et al., 2016).

## Hijacking Cellular Lipid Sources

As well as upregulating lipogenesis, viral infection can also redirect host lipids from other organelles for viral replication and connections with host organelles at membrane contact sites (MCSs) have been implicated in the formation of the RO (Table 1). A recent BioID study has identified several putative interactions between viral proteins and host MCS-localizing proteins, including lipid transfer proteins (St-Germain et al., 2020) (LTPs, see Box 1).

**TABLE 1:** Viral DMVs with known host organelle MCSs or lipid transport mechanisms.

Virus	RO^f^ (primary membrane source)	RO:host organelle contacts^g^	Host MCS/lipid transfer proteins^h^	Lipids^i^
Hepatitis C virus^a^	DMV (ER) (Romero-Brey et al., 2012)	ER and lipid droplets (Romero-Brey et al., 2012) and endosomes (Stoeck et al., 2018)	PI4KIIIβ/PI4KIIIα (Berger et al., 2009), OSBP (Wang et al., 2014), VAP (Paul et al., 2013), STARD3, PITPNM1/Nir2 and NPC1 (Stoeck et al., 2018), ORP1 and CERT (Stoeck et al., 2018)	PIP (Reiss et al., 2011), Cholesterol (Stoeck et al., 2018), PIP2 (Cho et al., 2015), PA (Mingorance et al., 2018), SL (Sakamoto et al., 2005; Hirata et al., 2012), FA (Khan et al., 2014)
Poliovirus^b^	Single-membrane tubules/DMV (Golgi) (Belov et al., 2012)	ER and lipid droplets (Laufman et al., 2019), mitochondria (Belov et al., 2012)	PI4KIIIβ (Belov et al., 2012; Arita, 2014), OSBP (Arita, 2014)	PIP cholesterol (Ilnytska et al., 2013), PL (Banerjee et al., 2018; Viktorova et al., 2018), FA (Viktorova et al., 2018)
Coxsackievirus B3^b^	Single-membrane tubules/DMV (ER/Golgi) (Limpens et al., 2011)	Lipid droplets (Melia et al., 2019)	PI4KIIIβ, OSBP (Lanke et al., 2009)	Cholesterol (Albulescu et al., 2015)
Aichi virus^b^	Unknown	ER (Ishikawa-Sasaki et al., 2018)	OSBP, PITPNB, VAP, PI4KIIIβ (Ishikawa-Sasaki et al., 2018)	PIP (Ishikawa-Sasaki et al., 2014), Cholesterol (Ishikawa-Sasaki et al., 2018)
Rhinovirus^b^	Multimembrane vesicles (Golgi) (Roulin et al., 2014)	Lipid droplets and ER (Roulin et al., 2014)	PI4KIIIβ (Roulin et al., 2014), OSBP, PITPβ, VAP (Roulin et al., 2014)	PI4P, Cholesterol (Roulin et al., 2014)
Encephalomyocarditis virus^b^	Single membrane tubules/DMV (ER) (Gazina et al., 2002; Melia et al., 2019)	ER	PI4KIIIα, OSBP (Dorobantu et al., 2015)	PIP (Dorobantu et al., 2015), cholesterol (Ilnytska et al., 2013; Albulescu et al., 2015; Dorobantu et al., 2015)
Norovirus^b^	DMV (ER) (Doerflinger et al., 2017)	Lipid droplets and endosomes (Doerflinger et al., 2017)	VAP (McCune et al., 2017)	Unknown
SARS CoV^c^	DMV (ER) (Snijder et al., 2006; Knoops et al., 2008)	Mitochondria (Snijder et al., 2006; Knoops et al., 2008; Snijder et al., 2020)	Unknown	Unknown
MERS-CoV^c^	DMV/CMs (ER?) (de Wilde et al., 2013)	Mitochondria (Snijder et al., 2020)	Unknown	LPL (Muller et al., 2018)
Human coronavirus-229E^c^	DMV (ER?) (Snijder et al., 2020)	(ER)	Unknown	LPL and ceramide (Muller et al., 2018)
SARS-CoV2^c^	DMV (ER?) (Wolff et al., 2020a)	Peroxisomes and mitochondria (Cortese et al., 2020), LDs, and lysosomes (Figure 1)	Unknown	Unknown
Porcine reproductive and respiratory syndrome virus^d^	DMV (ER) (Zhang et al., 2018)	Unknown	Unknown	FA (Long et al., 2019), Cholesterol (Jeon and Lee, 2017)
Berne virus^e^	DMV (?) (vila-Perez et al., 2016)	ER and mitochondria (vila-Perez et al., 2016)	Unknown	Unknown

*Viruses from the families: ^*a*^Flaviviridae, ^*b*^Picornaviridae, ^*c*^Coronaviridae, ^*d*^Arteriviridae, and ^*e*^Tobaniviridae. ^*f*^Viral replication organelle structures. ^*g*^Observed contacts between viral replication organelles and host organelles. ^*h*^Host factors localizing to membrane contact sites or involved in lipid transfer that appears to be involved in viral replication. ^*i*^Host lipid species implicated in viral replication. CM, Convoluted membrane; DMV, double-membrane vesicle; ER, endoplasmic reticulum; FA, fatty acids; LPL, lysophospholipids; MERS-CoV, Middle East respiratory syndrome-related coronavirus; PA, phosphatidic acid; PIP, phosphatidylinositol-phosphate; PIP2, phosphatidylinositol bisphosphate; PLs, phospholipids; SARS-CoV, severe acute respiratory syndrome coronavirus; SL, sphingolipids.*

### ER to DMV Lipid Transport

Golgi-localized oxysterol-binding protein (OSBP) mediates transport of newly synthesized cholesterol from the ER to the Golgi at MCS, in a counter-exchange mechanism whereby the phospholipid PI4P is transferred from the Golgi to the ER (Mesmin et al., 2013) (see Box 1). This PI4P-cholesterol counter flux mechanism can be usurped to support viral replication. Several diverse (+)RNA viruses including poliovirus (Arita, 2014), HCV (Wang et al., 2014), rhinovirus (Roulin et al., 2014), Aichi virus (Ishikawa-Sasaki et al., 2018), and encephalomyocarditis virus (Dorobantu et al., 2015) hijack OSBP by recruiting PI4-kinase (PI4K) to their RO. Viral PI4K recruitment enriches the RO with PI4P, increasing OSBP recruitment and therefore PI4P-cholesterol counter-exchange at ER contacts to supply cholesterol for viral replication.

**Box 1. Lipid transport at membrane contact sites.**

Membrane contact sites (MCSs) are formed when membranes of different organelles are tethered (Eisenberg-Bord et al., 2016) in very close proximity (typically < 30 nm) but without membrane fusion (Scorrano et al., 2019). MCSs occur between most pairings of cellular organelles and have a range of functions important for the regulation of cellular physiology. MCSs provide sites of interorganellar communication, including lipid and calcium exchange, as well as regulating organelle dynamics and positioning (Bohnert, 2020; Prinz et al., 2020; Silva et al., 2020). As such, MCSs are dynamic, with their extent finely tuned to meet the needs of the cell or organelle. An example of this is the expansion of ER–endosome MCSs during maturation of the endosome (Friedman et al., 2013), coordinating endocytic fission events for recycling/retrograde transport (Rowland et al., 2014; Allison et al., 2017; Hoyer et al., 2018), with endosome positioning (Raiborg et al., 2016; Cabukusta and Neefjes, 2018; Di Mattia et al., 2019), signaling regulation (Eden et al., 2010; Palande et al., 2011; Jongsma et al., 2016; Hong et al., 2017), and lipid and calcium exchange (van der Kant and Neefjes, 2014; Atakpa et al., 2018; Lee and Blackstone, 2020; Martello et al., 2020).

Specific proteins, or more commonly protein complexes, can bridge the two opposing membranes and populate and regulate MCSs (Scorrano et al., 2019). One type of protein enriched at MCSs are LTPs, which facilitate non-vesicular lipid transport between the two membranes (Wong et al., 2019). These proteins have domains with a hydrophobic cavity or groove that can accommodate one or more lipid monomers, protecting the hydrophobic element from the cytosol. Many LTPs undergo rapid conformational changes in response to the lipid environment and often reside on more than one organelle, with the ability to bridge MCSs and transport lipids against their concentration gradient according to the needs of the cell. Recent developments in the field have identified lipid counter-exchange as a common way in which LTPs operate at MCSs.

OSBP is a soluble mammalian LTP that usually localizes to ER–Golgi MCSs. This localization is a result of simultaneous recruitment to the ER membrane via interaction with ER-residing VAP proteins and to the Golgi membrane by the enrichment of PI4P lipids at the membrane by Golgi-specific PI4KIIIβ. At ER–Golgi MCSs, OSBP mediates transport of newly synthesized cholesterol from the ER to the Golgi. This cholesterol transfer occurs against its concentration gradient, driven by a counter-exchange mechanism with the phospholipid (Mesmin et al., 2013). The generation of PI4P at the Golgi by PI4KIIIβ at the Golgi membrane and the consumption of PI4P at the ER membrane by the phosphatase Sac1 maintain a high concentration gradient of PI4P, allowing the transport of PI4P down its gradient from Golgi to the ER and the counter-transport of cholesterol from ER to Golgi.

A mechanism of regulation of plasma membrane phospholipids by MCS has also been proposed. Phosphorylation of PI4P increases the PI(4,5)P_2_ composition of the plasma membrane. When PI(4,5)P_2_ is elevated, an integral ER membrane protein, ORP8, is recruited to ER–plasma membrane MCSs. Together with another ER-resident OSBP, ORP5, ORP8 at MCSs mediates transport of ER-derived PS to the plasma membrane in exchange for plasma membrane-derived PI4P to limit PI(4,5)P_2_ production and maintain homeostatic levels of phospholipids at the plasma membrane (Sohn et al., 2018).

PI4Ks include four isoforms in human cells. Enteroviruses such as coxsackievirus recruit PI4KIIIβ to their RO (Hsu et al., 2010) to generate PI4P and recruit OSBP. This exploitation of MCS machinery to drive OSBP/PI4P-dependent cholesterol transport from the ER to the viral ROs plays an important role in efficient viral replication as PI4KIIIβ kinase inhibition or OSBP depletion significantly reduced viral RNA synthesis (reviewed in Altan-Bonnet, 2017). Alternatively, other viruses, including cardiovirus and HCV, recruit OSBP using PI4KIIIα (usually found at ER-PM contact sites) (Dorobantu et al., 2015). Viral protein NS5A was found to bind and activate PI4KIIIα, raising PI4P levels on the viral DMVs (Berger et al., 2011; Reiss et al., 2011), and depletion of PI4KIIIα or PI4P inhibited HCV replication. Interestingly, for HCV and cardiovirus, the normal RO structure was also altered with OSBP/PI4K depletion, suggesting that the action of OSBP is important for the structural characteristics of DMVs. SARS-CoV-2 nsp4 was recently found to interact with OSBP and PI4KIIIβ in a BioID study (St-Germain et al., 2020), suggesting that SARS-Cov-2 may also utilize OSBP-mediated lipid exchange for cholesterol enrichment.

### Golgi to DMV Lipid Transport

+RNA viruses can also utilize other PI4P-binding proteins. The PI4P effector, four-phosphate adaptor protein 2 (FAPP2), a Golgi-localizing LTP, is also involved in HCV replication and is speculated to transport glycosphingolipids to the RO (Khan et al., 2014). Depletion of FAPP2 caused massive inhibition (>100-fold) of viral RNA replication. Both the PI4P-binding PH domain, which is thought to localize FAPP2 to the Golgi, and its glycolipid transport domain were found to be important for HCV replication. Similarly, depletion of another Golgi PI4P-binding protein, GOLPH3, also inhibited HCV secretion (Bishe et al., 2012).

### Lipid Droplets (LDs)

In addition to activation and hijacking of host lipid transport mechanisms, it is also in the virus’s interests to simultaneously downregulate lipid storage. Cholesterol is esterified in the ER prior to transport for storage in LDs, which are dynamic organelles where neutral lipids are stored, synthesized, and mobilized according to cellular requirements. Enteroviruses inhibit cholesterol esterification, preventing its transport to LDs, thereby increasing the availability of cellular cholesterol for use in viral ROs. This corresponds with reduced LDs (Ilnytska et al., 2013). Congruent with viral exploitation of cellular lipid stores, enterovirus infection induces MCS formation between ROs and existing LDs that provide platforms for transport of fatty acids to the RO. Electron microscopy (EM) studies have shown that ROs of rhinovirus, poliovirus, and coxsackievirus form MCSs with LDs (Roulin et al., 2014; Laufman et al., 2019; Melia et al., 2019), and similarly, we have observed DMV:LD contacts in cells infected with SARS-CoV-2 (Figure 1). In contrast to enterovirus infection, however, LDs and lipolysosomes were increased in SARS-CoV-2–infected cells, but not following SARS-CoV infection (Nardacci et al., 2020). Another intriguing observation was an increase in MCSs between mitochondria and LDs, possibly in response to dysregulation of host organelles by viral infection.

Importantly, inhibition of LD lipolysis was found to disrupt both RO formation and enterovirus replication, suggesting an important role for these LDs in the provision of lipids for RO biogenesis (Laufman et al., 2019). Indeed, a number of studies have implicated LD-derived lipid transport to ROs as a mechanism to support poliovirus replication. Fluorescently labeled fatty acids from LDs were transported to polioviral ROs (Laufman et al., 2019), and LD-derived phospholipids are required for poliovirus replication (Viktorova et al., 2018; Laufman et al., 2019). Lipases are recruited to LDs in poliovirus-infected cells to mobilize and release free fatty acids, and the poliovirus protein 3A, which localizes to ROs, was found to interact with LD-associated lipases, inhibition of which prevented the development of ROs (Laufman et al., 2019).

HCV infection also causes membrane rearrangements around LDs, increasing contacts between LDs and cisternae membranes (Miyanari et al., 2007). HCV capsid protein associates with LDs and recruits non-structural proteins and replication complexes to LD-associated membrane (Miyanari et al., 2007).

While the molecular architecture of DMV:LD contact sites has not been characterized, similar mechanisms to those operating at the ER:LD interface likely tether these contacts and provide sites for lipid transport to the DMV. The integral ER protein seipin facilitates ER to LD trigyceride flow at lipidic bridges that form between the two organelles, where the LD phospholipid monolayer is continuous with the outer leaflet of the ER membrane (Salo et al., 2019). Several other proteins have been implicated in tethering LD contact sites, including the triglyceride synthesis enzyme DAG acyl transferase 2 (DGAT2) (Xu et al., 2012), Rab18 (Xu et al., 2018), and Snx14 (Datta et al., 2019). Interestingly, an interaction between the LD tethering protein Rab18 and viral nsp7 was identified in a SARS-CoV-2 virus–host protein interactome (Gordon et al., 2020). When viewed together with the known role of LDs in viral replication and the extensive contact between DMVs and LDs in SARS-CoV-2–infected cells, these findings collectively provide compelling evidence of coronavirus appropriation of LD contact sites machinery for the biogenesis/maturation of its RO.

### Endosome to DMV Lipid Transport

Dietary cholesterol, packaged into lipoproteins, enters the cell through the endocytic pathway, which can be targeted by viruses as an alternative source of lipids. Several viruses enrich their RO with PI4P by PI4K recruitment. However, PI4K also directly interacts with endosomal Rab11 (Burke et al., 2014) and can recruit Rab11-positive recycling endosomes to ROs for cholesterol exchange (Ilnytska et al., 2013; Albulescu et al., 2015).

Reasoning that DMV cholesterol enrichment likely depends on host LTPs, one study set out to establish whether any known LTPs are required for HCV replication. Several HCV dependency late endosomal sterol-binding proteins were identified, including STARD3, OSBP1, and NPC1, all of which have roles at MCSs (Salvador-Gallego et al., 2017). A follow-up study provided further evidence that viruses sequester endocytic organelles to provide the sterols necessary for viral replication. A role for the late endosomal sterol-binding protein NPC1 in mediating recruitment of endosomal cholesterol to ROs via MCSs with late endosomes/lysosomes in cells infected with HCV was demonstrated (Stoeck et al., 2018). A recent proximity labeling BioID study identified SARS-CoV-2 protein crosstalk with several lipid transport and MCS proteins, including NPC1, StARD3, and MOSPD2 (St-Germain et al., 2020). However, while NPC1 mediates transport of low-density lipoprotein (LDL)–derived cholesterol from late endosomes/lysosomes to the ER, STARD3 transports newly synthesized cholesterol in the opposite direction, from the ER to the endosome (Wilhelm et al., 2017). It is, therefore, hard to envisage a role for STARD3 as an LTP that enriches DMVs with endosomal sterol, and a recent study screening a CRISPR library for proteins required for SARS-CoV-2 infection found that neither endosomal STARD3 nor one of its ER-localized binding partners, MOSPD2 (Di Mattia et al., 2018), mediates viral infection or virus-induced cell death (Schneider et al., 2020). Indeed, a second genome-wide CRISPR screen identified STARD3 STARD3NL and MOSPD2 as negative regulators of SARS-CoV2 infection (Schneider et al., 2020). However, consistent with a role for DMV:endosome MCSs in viral replication, a study mapping SARS-CoV-2 virus–host protein interactions identified several Rabs interacting with a viral protein (nsp7) (Gordon et al., 2020), including Rab7, which is important for endocytic traffic and ER–endosome contact site formation (Martello et al., 2020). Rab8, which promotes recycling of cholesterol-rich endosomes to the plasma membrane (Kanerva et al., 2013), was also found to interact with nsp7, suggesting that Rab8-dependent cholesterol transport may be diverted away from the plasma membrane in infected cells to contribute to the DMV cholesterol supply. That MCSs form between DMVs. and endocytic organelles (e.g., DMV:lysosome MCSs in Figure 1) further support the notion that SARS-CoV-2 can exploit MCS machinery to sequester LDL-derived lipids for its ROs.

## Manipulation of Mitochondria to Evade the Immune Response

In order to proliferate, viruses must survive the host’s immune response. Coronaviruses, including SARS-CoV-2, have evolved extensive measures to dampen the mechanism of innate sensing that cells use to sound the infection alarm. Multiple coronavirus proteins inhibit the production of interferons (IFNs) and cytokines from infected cells, thereby aiding viral replication. As discussed above, DMV formation offers protection for viral RNA replication, but viruses take further immune evasion measures, to actively disrupt the host innate immunity.

One intriguing aspect of innate immune sensing is the use of membrane surfaces for construction and regulation of many of the protein complexes responsible for determining signaling outputs. Mitochondria, in addition to their key role in cellular metabolism, are also important in the regulation of innate immunity in this context as they provide a platform for signaling complexes that drive IFN production and cell death. During infection, recognition of viral RNA by cytosolic pattern recognition receptors (PRRs) initiates oligomerization of the signaling adaptor mitochondrial antiviral signaling (MAVS), which in turn stimulates recruitment and activation of transcription factors that induce type I and type III IFN and proinflammatory cytokine production. There is a growing body of evidence that membrane reorganization and interactions help to orchestrate these events to promote appropriate antiviral responses. Sensing of intracellular RNA by PRRs results in extensive mitochondrional remodeling via regulation of mitochondrial fission through dynamin-like protein 1 (Drp1) (Chen et al., 2020) and fusion through mitofusin 1 (Mfn1) (Castanier et al., 2010). In non-infected cells, both fission and fusion events are defined by ER–mitochondria MCSs (Abrisch et al., 2020): interaction between mitochondrial Drp1 and ER-localized syntaxin 17 at MCSs promotes Drp1-mediated mitochondrial fission (Arasaki et al., 2015), whereas Mfn2 localizes to ER:mitochondria MCSs at sites of mitochondrial fusion (Abrisch et al., 2020). As discussed below, as both Drp1 and Mfn2 can be depleted by viral proteins, MCS disruption may contribute to mitochondrial remodeling in infected cells. Equally, virus infection can result in extensive mitochondrial damage, release of mtDNA, and subsequent activation of stimulator of IFN genes (STING)–dependent DNA sensing PRRs that also drive IFN-I transcription (Schoggins et al., 2014). MAVS and STING are both membrane-associated adaptor proteins that accumulate large signaling complexes on organelle surfaces and potentially at MCSs.

Extensive crosstalk between mitochondria and the ER at mitochondria-associated membranes (MAMs) is important for the regulation of mitochondrial function and signaling pathways involved in numerous cellular processes including inflammation and apoptosis (reviewed in Missiroli et al., 2018) and can be targeted by +RNA viruses to promote viral replication. Using time-lapse microscopy, the HIV-1 viral protein R (Vpr) was shown to be transported from the ER to mitochondria at MAMs (Huang et al., 2012). Vpr expression resulted in reduced levels of both Mfn2 and Drp1 and increased OMM permeability. Similarly, infection with dengue virus also resulted in reduced mitochondrial Mfn2 and Drp1, as well as elongated mitochondria due to a loss of Drp1-mediated fission. Reduced fission may advantage the virus through the associated impaired clearance of dysfunctional mitochondria potentially containing viral RNA or protein. Interestingly, dengue virus infection induced the formation of nsp4-containing ROs that were in close contact with mitochondria, possibly at the expense of MAM formation, as ER–mitochondria interactions appeared to be reduced. Thus, it seems likely that viral proteins targeted to the ER, where the RO originates, may also hijack interactions with mitochondria to enable disruption of MAVS immune signaling pathways. Indeed, dengue Ns4A/B proteins inhibit viral RNA sensing, blocking activation of Tank-binding kinase 1 (TBK1) downstream of MAVS (Dalrymple et al., 2015).

Loss of Drp1 was also observed in cells infected with coronavirus. A SARS-CoV protein, open reading frame-9b (ORF9b), was found to localize to mitochondria and promote ubiquination and proteosomal degradation of both Drp1 and MAVS protein, resulting in elongated mitochondria, increased viral replication and inhibition of immune activation. Mitochondrial ORF9b mediates recruitment of the E3 ligase AIP4, to promote MAVS polyubiquitination. Reducing MAVS degradation through AIP4 depletion or disruption of AIP4 mitochondrial recruitment reversed the Orf9b-mediated suppression of antiviral immune response (Shi et al., 2014). DMV:mitochondria MCSs are common to Vero-E6 cells infected with SARS-CoV (Snijder et al., 2006) or A549 lung carcinoma epithelial cells infected with SARS-CoV-2 (Figure 1), and a recently published interactome suggests that SARS-CoV-2 also targets mitochondria, identifying interactions between viral Nsp4 and host mitochondrial TIMM complex, Nsp8, and mitochondrial ribosome proteins, and ORF9C and mitochondrial electron transport proteins (Gordon et al., 2020). Indeed, SARS-CoV-2 ORF9b also localizes to mitochondria and suppresses type I IFN responses by targeting mitochondrial TOM70 (Jiang et al., 2020). Interestingly, TOM70 overexpression can overcome type I IFN inhibition. Moreover, STING has also been implicated in SARS-CoV-2 innate sensing (Neufeldt et al., 2020), suggesting mtDNA release following SARS-CoV-2–induced mitochondrial damage.

Despite the nomenclature, MAVS can be found on peroxisomes, as well as mitochondria, with peroxisome-targeted MAVS producing a rapid transient response to influenza virus infection, complementing the delayed and stable response produced by mitochondria-targeted MAVS (Dixit et al., 2010). The influenza nsp, NS1, plays important roles in resisting host cell immune responses. NS1 was found to interact with a peroxisome-targeted host protein NS1-interactor (NS1-I) (Wolff et al., 1996), implicating peroxisome crosstalk in viral immune evasion. Crosstalk between HIV and peroxisomes was also found to contribute to dampening of the immune response, with interaction between HIV Nef and a peroxisomal thioesterase associated with CD4 downregulation and enhanced viral infectivity (Cohen et al., 2000). Roles for peroxisome proteins contributing to SARS-CoV-2 immune evasion have not yet been established, but clustering of peroxisomes in close proximity to DMVs was identified in cells infected with SARS-CoV-2 (Cortese et al., 2020). Moreover, a recent proximity labeling BioID study identified SARS-CoV-2 nsp4 association with VAP and ACBD5 (St-Germain et al., 2020), putative ER:peroxisome MCS proteins (Costello et al., 2017). The functional significance of this peroxisome association with DMVs is not yet known, but mechanisms for the prevention of oxidative damage to viral RNA or for peroxisome-mediated lipid metabolism in viral replication were proposed (Cortese et al., 2020).

## Discussion

The morphology of enterovirus ROs develops and changes during the course of viral infection, suggesting that ROs are dynamic and undergo a form of maturation. Polio and coxsackie ROs start as tubules that transform into DMVs, which are then enwrapped with more tubules to form multilamellar structures at later stages of infection (Limpens et al., 2011; Belov et al., 2012). For coronaviruses, the exact mechanism for DMV formation is as yet unconfirmed, but the number of DMVs, as well as the extent of surrounding convoluted membranes, increases during infection, and vesicle packs form late in the infection. The distribution and morphology of the RO change over the course of infection, which could in itself induce changes in its interaction with other organelles. Depletion of lipids from host organelles such as LDs could also be a factor in changes in MCS occurrence. Taken together, evidence suggests that MCSs between ROs and host cell organelles are regulated for the provision of specific lipids to the RO during infection, as required for replication of +RNA viruses. The importance of viral exploitation of host MCS machinery for biogenesis of the RO is further substantiated by a recent BioID proximity labeling study identifying an association between SARS-CoV-2 proteins and proteins functioning at ER contact sites with a variety of cellular organelles, including the plasma membrane (Gramd1a/b, eSyt, STIM1, ORP8), endo/lysosomes (NPC1, ORP1L, STARD3, PDZD8, ORP8, VAP, MOSPD2, Gramd1b), LDs (ORP2, VPS13A), mitochondria (ORP8, VPS13A, PDZD8), Golgi (VAP, ORP9/10), and peroxisomes (VAP, ABCD5) (St-Germain et al., 2020). As well as physically protecting viral RNA from host immune responses, host membrane rearrangements, often exploiting MCS machinery, can also compromise innate sensing to evade mitochondrial mediated immune response and safeguard viral replication.

## Data Availability Statement

The raw data supporting the conclusions of this article will be made available by the authors, without undue reservation.

## Author Contributions

All authors listed have made a substantial, direct and intellectual contribution to the work, and approved it for publication. Images for Figure 1 were provided by JE.

## Conflict of Interest

The authors declare that the research was conducted in the absence of any commercial or financial relationships that could be construed as a potential conflict of interest.
